# Standardized Response Assessment in Patients with Advanced Cholangiocarcinoma Treated with Personalized Therapy

**DOI:** 10.3390/jpm14121143

**Published:** 2024-12-06

**Authors:** Stephan Ursprung, Wolfgang Thaiss, Janina Beha, Yvonne Möller, Nisar P. Malek, Meinrad Beer, Verena I. Gaidzik, Thomas Seufferlein, Ambros J. Beer, Konstantin Nikolaou, Christian Philipp Reinert

**Affiliations:** 1 Department of Radiology, University Hospital Tuebingen, 72076 Tübingen, Germany; 2 Department of Nuclear Medicine, Ulm University Medical Center, 89081 Ulm, Germany; 3 Department of Radiology, Ulm University Medical Center, 89081 Ulm, Germany; 4 Center for Personalized Medicine, University Hospital Tuebingen, 72076 Tübingen, Germany; 5 Department of Internal Medicine I, University Hospital, Eberhard-Karls University, 72076 Tübingen, Germany; 6 Cluster of Excellence, Image Guided and Functionally Instructed Tumor Therapies, Eberhard-Karls University, 72076 Tübingen, Germany; 7 German Cancer Consortium (DKTK) and German Cancer Research Center (DKFZ), 69120 Heidelberg, Germany; 8 National Center for Tumour Diseases SouthWest: Tuebingen-Stuttgart/Ulm, 89070 Ulm, Germany; 9 Innovative Imaging in Surgical Oncology, Ulm University Hospital, 89070 Ulm, Germany; 10 Department of Internal Medicine I, Ulm University Hospital, 89081 Ulm, Germany; 11 Center of Personalised Medicine, University Hospital Ulm, 89081 Ulm, Germany; 12 Department of Internal Medicine III, Ulm University Hospital, 89081 Ulm, Germany

**Keywords:** cholangiocarcinoma, molecular tumor board, personalized cancer care, imaging

## Abstract

**Background/Objectives**: Current guidelines recommend Cisplatin/Gemcitabine/Durvalumab as first-line treatment for inoperable or recurrent cholangiocarcinoma (CCA). Molecular tumor boards (MTB) have the expertise to support organ-specific tumor boards with evidence-based treatment recommendations for subsequent lines of treatment, based on genomic tumor data and scientific evidence. This study evaluates the adoption of an MTB at a comprehensive cancer center in Germany and whether actionable genetic alterations are associated with specific imaging phenotypes. **Methods**: Patients with CCA referred to MTB were enrolled from May 2019 to September 2021. For comparison, a cohort of patients from a second center was included. Data on treatment recommendations, regimens, and survival were collected from prospective registries. Baseline and follow-up contrast-enhanced CT were analyzed according to RECIST 1.1. The chi-square test and *t*-test were used to compare categorical and continuous variables. **Results**: 583 patients were referred to the MTB, and 92 patients (47 female/51%) with a mean age of 60.3 ± 11.2 were referred for CCA treatment. 65/92 patients harbored 1–3 targetable mutations. Liver metastases were more frequently observed in patients with targetable mutations (84% vs. 62%). Metastasis to the liver and lung was associated with increased sums of diameters (93 mm and 111 mm vs. 40/73 mm in patients with no liver/lung metastasis). The number of metastases in individual organs was unrelated to treatment targets. Follow-up was available for 25 patients with a median time until imaging progression of 23 weeks. Progression occurred as target progression in 63%, nontarget progression in 13%, and appearance of new lesions in 63%. **Conclusions**: Most patients with CCA harbored targetable mutations, some were related to disease patterns on imaging. The pattern of treatment response and progression was as diverse as the metastatic spread.

## 1. Introduction

Biliary tract cancers account for 10–15% of all primary liver cancers globally and up to 80% regionally in southeast Asia [1]. In 2017, more than 200,000 patients were diagnosed with biliary tract cancers globally, accounting for almost 3,500,000 disability-adjusted life years [2]. Chronic liver disease, including cirrhosis and hepatitis, liver flukes, primary sclerosing cholangitis, and hepatolithiasis, are established risk factors for cholangiocarcinoma (CCA) [3,4]. CCA is significantly more incident in southeast Asia, reaching age-standardized incidence rates of 85/100,000 in North East Thailand, compared to western populations (2,1-3,3/100,000) [4,5]. Liver fluke infestation is thought to be a major contributor to disparate incidence rates.

The treatment of biliary tract cancers remains challenging, both in patients suitable for surgery and those undergoing systemic treatment. A 5-year recurrence-free survival remains approximately 2–39% following resection with curative intent [6]. Simultaneously, overall survival remains poor, with a 5-year overall survival of 7–20% [7]. Advanced disease stages, defined as locally advanced when resection is no longer possible and metastatically advanced in the presence of distant metastases, are particularly challenging to treat. Untreated, median overall survival may be as low as 3.9 months in this population [8]. In the past decade, cytotoxic chemotherapy with Gemcitabine/Cisplatin was the recommended first-line treatment for patients with advanced disease or unresectable recurrence, and no standardized second-line treatment was available [9,10].

The introduction of immunochemotherapy with cisplatin, gemcitabine, and durvalumab as the recommended first-line treatment induced a considerable shift in the treatment landscape. The combination treatment shows advantages over chemotherapy, with a median overall survival of 12.9 months vs. 11.3 months and a higher response rate of 26.7% vs. 18.7% [11]. Furthermore, the guidelines now recognize that almost 40% of all biliary tract cancers harbor actionable genetic alterations and recommend genetic testing as a standard of care for second-line treatment and beyond. Examples of actionable genetic alterations are fusions and rearrangements of the fibroblast growth factor receptor 2 (FGFR 2). Mutated FGFR 2 is thought to be a driver of CCA with enhanced signaling leading to increased proliferation, invasion, and angiogenesis [12]. Three selective inhibitors of FGFR 1–3, pemigatinib, infigratinib, and futibatinib, have been developed and have shown therapeutic potential in FGFR-mutated CCA [13,14]. Gain of function mutations in isocitrate dehydrogenase 1 and 2 (IDH 1/2) are found in approximately 13% of intrahepatic CCA and promote the formation of the oncometabolite 2-hydroxyglutarate [15,16]. Ivosidenib is an inhibitor of IDH 1/2. In a placebo-controlled, phase III trial, ivosidenib has resulted in an increased overall survival of 10.3 months (95% confidence interval: 788-12.4 months) compared to 7.5 months (95% CI: 3.8–7.6 months) with placebo [17].

With more treatment options and a disease that remains challenging to treat, imaging follow-up is crucial in assessing treatment effectiveness and detecting potential complications [18]. The diagnosis of CCA also relies on imaging and histological analyses. The local tumor extent is determined using MRI, while distant staging is performed using CT. The role of [18F] fluorodeoxyglucose positron emission tomography (FDG-PET) in diagnosis and treatment planning has been investigated repeatedly. A recent meta-analysis found the sensitivity and specificity of [18F] FDG-PET for identifying a primary CCA of 91.7% and 51.3%, respectively. It calculated a pooled sensitivity and specificity for lymph node metastases of 88.4% and 69.1% and for distant metastases of 85.4% and 89.7%. The authors noted that [18F] FDG-PET prompted treatment changes in 15% of patients, primarily due to upstaging in 78% of cases. The frequency of treatment change was similar whether FDG-PET was performed before or after the surgical resection. [19]. Another recent study demonstrated that [18F] FDG-PET/CT affected the planned treatment and changed the therapeutic approach for 60.5% of CCA patients at various stages of diagnosis and treatment [20]. Consequently, [18F] FDG-PET may be a valuable tool for refining treatment decisions and confirming disease recurrence. However, it is not recommended for primary diagnosis, owing to its limited sensitivity to infiltrative CCA [10]. Its use is suggested after interdisciplinary consultation.

Several criteria for determining tumor activity exist. Nonetheless, evaluating cancer treatment response in solid tumors most commonly involves consecutive clinical or radiologic assessments of target lesions, where a significant reduction in measurable tumor size is recognized as meaningful [21]. However, altered response patterns to targeted therapy and immune checkpoint inhibitors mean that response criteria developed for cytotoxic chemotherapy may not universally apply to newer treatments. Recent studies have shown that physiological imaging features like perfusion may be earlier signs of treatment response than changes in tumor size and, alterations in response criteria are needed to accommodate treatment-specific responses like pseudoprogression in immune checkpoint inhibition [21,22]. Additionally, imaging plays a role in investigating adverse events, the spectrum of which is specific to the treatment [18].

In essence, CCA shows remarkable genetic diversity and poses challenges in diagnosis, treatment, and follow-up [23]. Similar to the genotype, the phenotype of CCA varies widely, both in terms of metastatic sites and metastatic patterns within organs. The question of how the genotype influences the metastatic phenotype has not been investigated. The timely identification of a tumor phenotype associated with aggressive disease when genomic data is not routinely available may influence patient management.

Here we investigate the imaging features of patients with advanced CCA referred to a molecular tumor board (MTB) at a comprehensive cancer center and review imaging responses to molecularly targeted treatments. The MTB provides treatment recommendations based on molecular tumor characteristics to organ-specific tumor boards once patients exhaust treatment options offered in clinical guidelines.

## 2. Materials and Methods

### 2.1. Patient Cohorts

Patients with advanced CCA referred to the MTB at a tertiary cancer center in Germany between May 2019 and September 2021 were retrospectively recruited into this study (92 patients). For the evaluation of imaging response to molecularly targeted treatments, a second cohort from another cancer center in Germany between May 2019 and September 2021 was included (16 patients). Prior ethical approval was sought from the institutional ethics committee (Project number: 110/2022BO2, date of approval: 23 February 2022). The requirement for informed consent was waived owing to the retrospective nature of the study.

The MTB kept a prospective, pseudonymized registry, including all patients referred for an MTB opinion and collected information on patient demographics, disease characteristics, prior treatment, molecular analyses, treatment recommendations, follow-up, and survival. The participants for this study were identified from this prospectively maintained registry, and all patients referred for the assessment of CCA were included. The database was last searched on 31 October 2022.

### 2.2. MTB Workflow and Molecular Profiling

**Figure 1:**
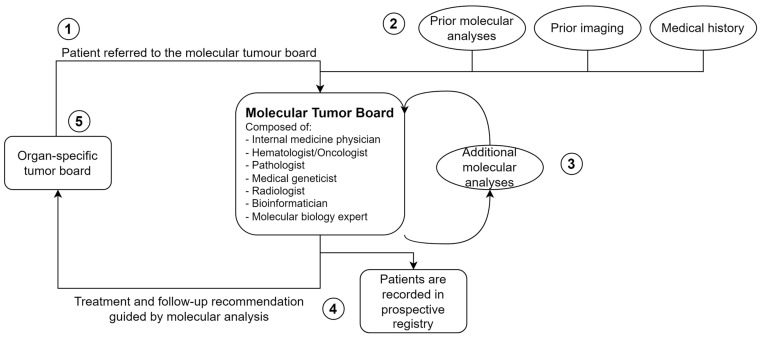
Workflow graph for the molecular tumor board (MTB). The molecular tumor board is composed of internal medicine, hematology and oncology, pathology, genetics, radiology, bioinformatics, and molecular biology experts. Depending on the tumor entity, additional relevant medical specialties are included. (1) Patients are referred to the MTB through the organ-specific tumor boards. (2) The MTB collects existing patient data, including molecular analyses and imaging. (3) Missing molecular analyses will be completed. (4) The MTB issues treatment and follow-up recommendations. Cases are recorded in a prospective registry. (5) Treatment recommendations are implemented by the referring organ-specific tumor board. The patient may be re-referred to the MTB upon disease progression.

As described above, the organ-specific MTB may refer patients to the MTB when treatment recommendations based on guidelines have been exhausted (Figure 1). The MTB consisted of hematologists, oncologists, pathologists, geneticists, and bioinformaticians in addition to the treating specialists. Molecular profiling, obtained through a tumor panel and transcriptome sequencing, and scientific evidence-informed personalized treatment recommendations. The MTB returned recommendations for future treatment options and personalized follow-up recommendations to the organ-specific tumor board. When patients on molecularly targeted treatment experienced disease progression, a re-referral to the MTB was possible.

### 2.3. Imaging Protocols

Baseline and follow-up scans of all patients were identified from the patient registry and retrieved from the institutional picture archiving and communication systems (PACS). Imaging studies were pseudonymized at the time of export from the PACS using the inbuilt function of Syngo.via (Siemens Healthineers, version VB60G). All patient-identifying information was removed from Digital Imaging and Communications in Medicine (DICOM) headers. Only the study date was preserved, enabling a temporal correlation between imaging studies and treatment information. Patients were excluded if the imaging follow-up was performed outside the institution’s healthcare network and was, therefore, unavailable for review.

Imaging was performed with portal venous phase contrast-enhanced multidetector CT (Siemens Somatom Definition AS, Somatom Force, Somatom Flash, or Naeotom Alpha), acquired 70 s after the injection of the contrast agent. The tube voltage and current were adapted to the patients’ body habitus (CARE kV and CARE Dose). Contrast agents (Imeron 350; Bracco Imaging or Ultravist 370, Bayer Vital) were dosed according to the patients’ body weights. Images were reconstructed in axial orientation with a slice thickness of 3 mm and in a soft tissue kernel. Some patients were referred with imaging from other institutions. Images reconstructed with a soft tissue kernel and a slice thickness ≤ 5 mm were used for analysis.

At the physician’s discretion and dependent on the tumor board’s recommendation, additional [18F] FDG-PET/CT or MRI were performed if CT did not accurately capture the tumor manifestations or where metabolic activity was thought to be of prognostic value. For [18F] FDG-PET/CT, patients were asked to fast for 6 h before the exam, and blood glucose levels were confirmed to be under 7.8 mmol/l. [18F] PET/CT was acquired on a Siemens Biograph mCT 128 slice scanner. Diagnostic CT scans were acquired in the portal venous phase (120 mL Ultravist, Bayer) at 120 kV using automatic exposure control (Care Dose). The diagnostic CT was used for attenuation correction. [18F] FDG-PET was performed as a static 3D acquisition 60 min after the i.v. application of 3 MBq/kg body weight. MRI of the liver and upper abdomen was acquired with a standardized protocol, including diffusion-weighted MRI and dynamic contrast-enhanced sequences.

### 2.4. Standardized Response Assessment

The institutional picture PACS was searched for imaging data of patients identified in the prospectively kept registries of the MTBs. The baseline examination was identified from the outcome letter of the MTB. The baseline examination and all subsequent cross-sectional imaging data were anonymized and exported from the PACS.

Treatment response was evaluated according to RECIST 1.1 [24] using MintLesion (Version 3.8.6, Mint Medical, Heidelberg, Germany). Tumors were outlined manually on the slice with the largest diameter, and the major and minor diameters were determined automatically. The sum of the relevant diameters (short axis diameter for lymph node metastases, long axis diameters for other metastases; SOD) was calculated and the response was classified according to the RECIST 1.1 criteria.

### 2.5. Patient Outcome

Patient data, including demographics, prior treatments, treatment recommendations, and administered treatments, were extracted from the patient registry. The evolution of the tumor burden was classified according to the RECIST 1.1 criteria, as described above. Information on adverse events was collected from the clinical information system.

### 2.6. Statistics

Statistical analysis was performed with the R language for statistical computing (version 4.2.3; R Foundation for Statistical Computing, Vienna, Austria [25]) using the ggplot 2 (version 3.4.1 [26]), survival (version 3.5–5 [27]), and survminer (version 0.4.9 [28]) packages. Data are represented using the mean and standard deviation for normally distributed data and the median and interquartile range for other quantitative data. Normality was assessed using the Shapiro–Wilk test. Chi-square tests were used to determine differences in the distribution of categorical data. Continuous variables were compared with the *t*-test when the variables were normally distributed; otherwise, the Mann–Whitney U test was used. Survival curves were compared using the log-rank test.

## 3. Results

### 3.1. Patient Characteristics

Between May 2019 and September 2021, 583 patients were referred to the MTB, of which 92 were referred for cholangiocarcinoma. The average age at baseline imaging was 60.3 ± 11.2 years. Additionally, 47/92 (51%) of the patients were female. Baseline imaging was available for 81 patients, on average, 10 weeks elapsed between baseline imaging and the tumor board recommendation (standard deviation: 5 weeks). The most common sites of metastasis were the liver, lymph nodes, lung, and peritoneum in 76%, 27%, 23%, and 22% of patients. Metastases to the pleura, the ovaries, the bone, the adrenals, and soft tissue were less frequently observed, each <15% (Supplementary Materials, Table S1).

A molecular target was identified using a tumor panel and transcriptome sequencing in most patients (61/92, 66%). Overall, targets relating to 14 treatment groups were identified (Table 1). The most commonly identified treatment options were FGFR inhibition (*n* = 13), poly (ADP-ribose) polymerase (PARP)-inhibition (*n* = 10), tyrosine kinase inhibition (*n* = 10), immune checkpoint inhibition (*n* = 6), and IDH-inhibition (*n* = 6). The remaining treatment options were identified less frequently: mitogen-activated protein kinase (MEK) inhibition (n = 3), phosphoinositide 3 (PI3)-kinase inhibition (n = 2), cyclin-dependent kinase (CDK)-4/6 inhibition, yes-associated protein (YAP) inhibition, ataxia telangiectasia and Rad3-related (ATR) inhibition, vascular endothelial growth factor receptor (VEGFR) inhibition, hepatocyte growth factor receptor (MET) inhibition, and B-Rapidly Accelerated Fibrosarcoma (BRAF inhibition (n = 1 each)). One patient could harbor several molecular targets.

### 3.2. Association of Imaging Features and Molecular Targets

The identification of molecular treatment targets was positively associated with metastasis to the liver. Liver metastasis was present in 84% of patients with a molecular target, whereas patients without a treatment target harbored liver metastasis in 62% (chi-squared test, X-squared = 3.82, degrees of freedom = 1, *p* = 0.05). Similarly, the presence of adrenal metastases was positively associated with targetable genetic variants of the PI-3-kinase (50%). Meanwhile, such mutations were only present in 1% of patients without adrenal metastases (chi-squared test, X-squared = 4.39, degrees of freedom = 1, *p* = 0.04). No other association between the localization or number of metastases and specific treatment recommendations was observed. The number of metastases in individual organs was unrelated to the presence of molecular treatment targets.

### 3.3. RECIST 1.1 Assessment and Molecular Targets

The mean SOD in the patient cohort was 81 mm (range: 0–274 mm; SD: 60 mm).

**Figure 2:**
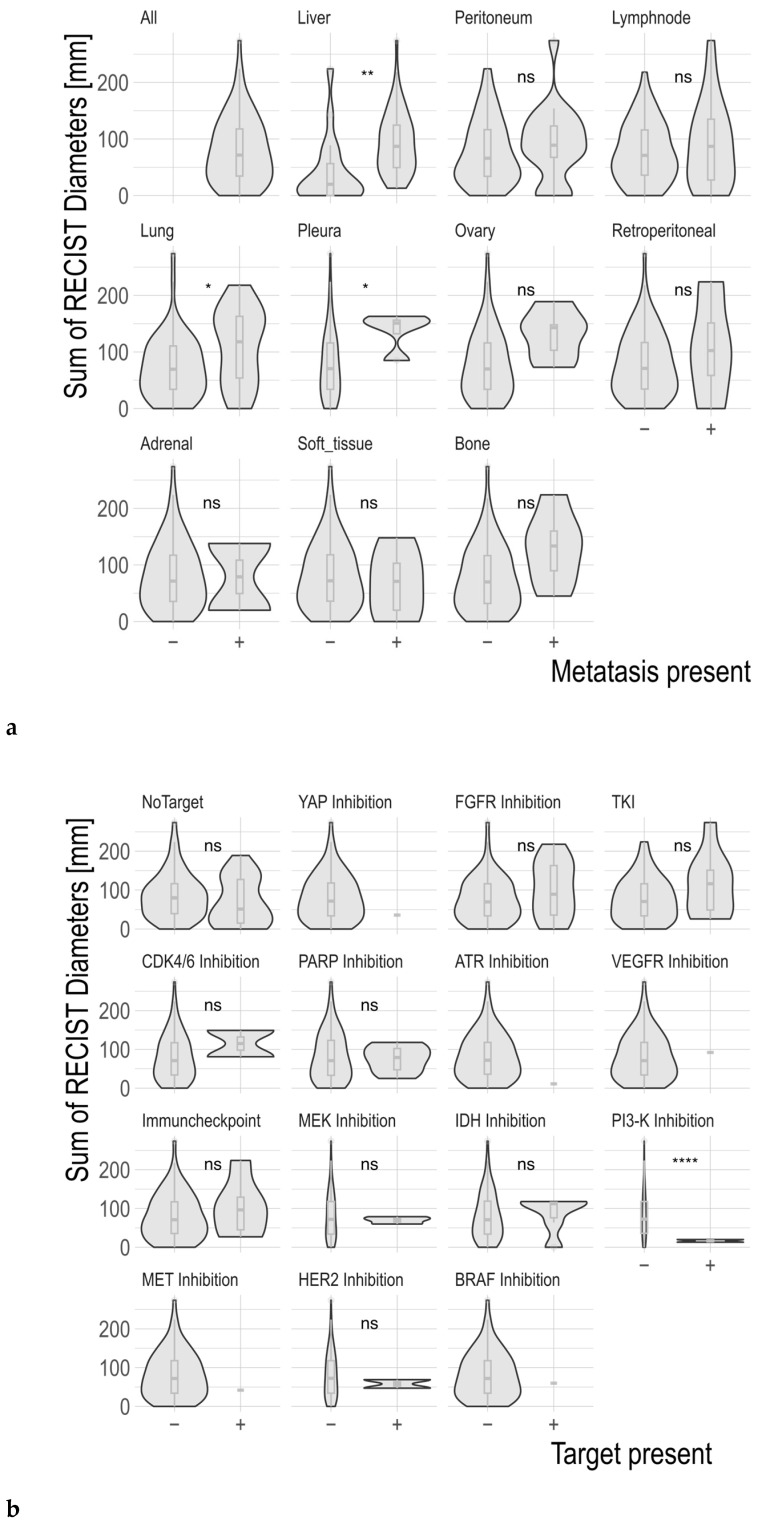
Comparison of the sum of diameters of all RECIST target lesions, stratified according to the presence of lesions in different organs (**a**) or the presence of specific treatment targets (**b**). Data are represented as violin plots and boxplots (hinges: 25th and 75th percentile, whiskers: highest and lowest value, no further than 1.5-times the interquartile range). ns: not significant, * *p* ≤ 0.05, ** *p* ≤ 0.01, and **** *p* ≤ 0.0001. ATR: ataxia telangiectasia and Rad3-related, BRAF: B-rapidly accelerated fibrosarcoma, CCA: cholangiocarcinoma, CDK: cyclin-dependent kinase, FGFR: fibroblast growth factor receptor, HER2: human epidermal growth factor receptor 2, IDH: isocitrate dehydrogenase, MEK: mitogen-activated protein kinase, MET: hepatocyte growth factor receptor, PARP: poly (ADP-ribose) polymerase, PI3: phosphoinositide 3, SD: standard deviation, TKI: tyrosine kinase inhibition, VEGFR: vascular endothelial growth factor receptor, YAP: yes-associated protein.

Patients with liver metastases had a higher tumor burden, approximated by a greater SOD, than those without (93 mm vs. 40 mm; *p* = 0.001). Similarly, patients with lung metastases had increased sums of diameter (111 mm vs. 73 mm; *p* = 0.04). Metastasis to the ovaries was borderline significantly associated with increased RECIST diameters (131 mm vs. 78 mm; *p* = 0.05; Figure 2a).

Some treatment targets showed an association with the tumor burden: patients who received a recommendation to undergo PI-3 kinase inhibition had a significantly shorter SOD (17 mm vs. 82 mm). None of the other molecular targets was associated with tumor diameters (Figure 2b).

### 3.4. Treatment Following MTB Referral

Sixty-one patients received treatment recommendations based on the tumor genotype. Some of those recommendations were off-label. Each patient harbored between one and three targetable molecular alterations. Twenty-two (36%) of these patients were treated with one of the recommended treatments during the follow-up period. From the second, external cohort of patients, another six patients could be included.

Nine patients underwent another line of cytotoxic chemotherapy before starting the personalized treatment. During the intermittent chemotherapy, six patients achieved disease stabilization, and three were progressive at the first follow-up. The median time to progression on imaging was 25 weeks.

### 3.5. Treatment Response

Twenty-eight patients commenced their recommended, molecularly defined treatment. Three patients did not survive until the first follow-up examination. Following the presentation in the tumor board, one patient was treated and followed up externally. (Supplementary Materials, Table S2 for treatment and molecular rationale).

Baseline and follow-up imaging in the molecular treatment line were acquired with CT (19/27 and 17/24) or [18F] FDG-PET/CT (8/27 and 7/24). Additional liver MRI was acquired in 3/27 patients at baseline and 3/24 during the follow-up. According to RECIST 1.1, one patient treated with pembrolizumab achieved a complete response. Five patients, treated with peptide vaccination, pembrolizumab, pemigatinib (2), or cabozantinib, achieved a partial response. The remaining patients experienced either disease stabilization or progressive disease at the first follow-up (Supplementary Materials, Table S3 for details on treatment response).

**Figure 3:**
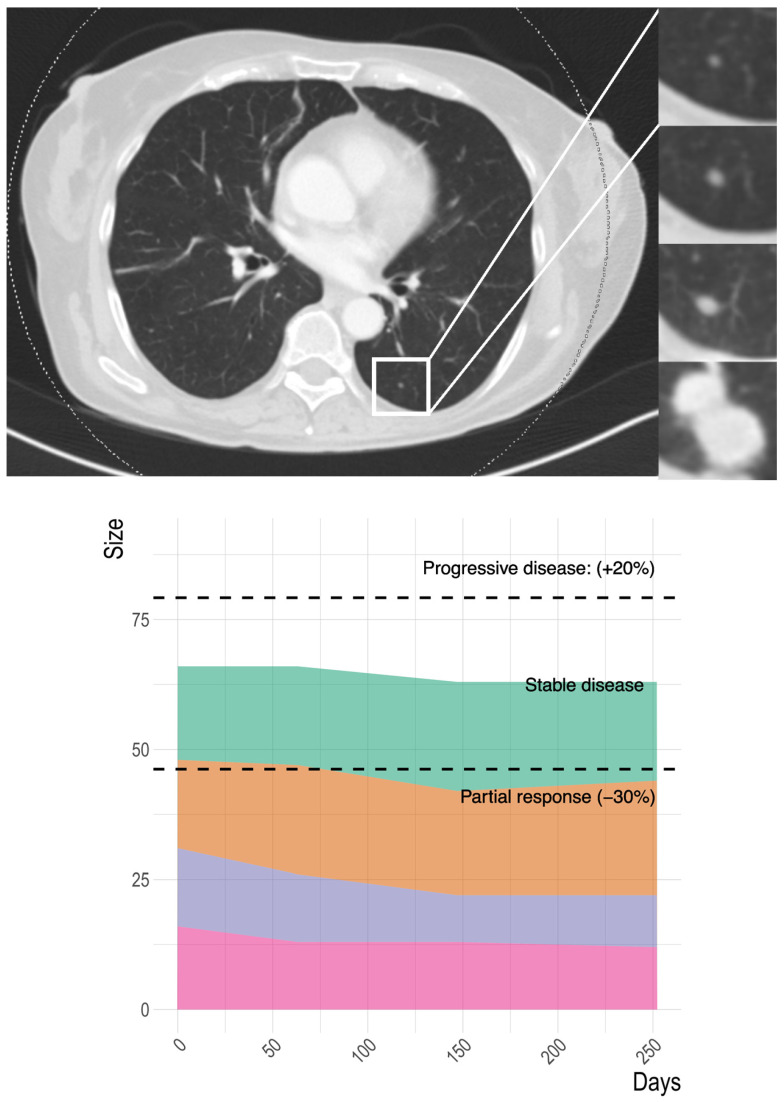
Example of a 64-year-old patient experiencing nontarget progression on FOLFOX treatment. A lung nodule, that was too small to serve as a RECIST target lesion at baseline, showed unambiguous progression at 36 weeks (**upper panel**). The target lesions remained stable over the duration of the treatment (**lower panel**).

**Figure 4:**
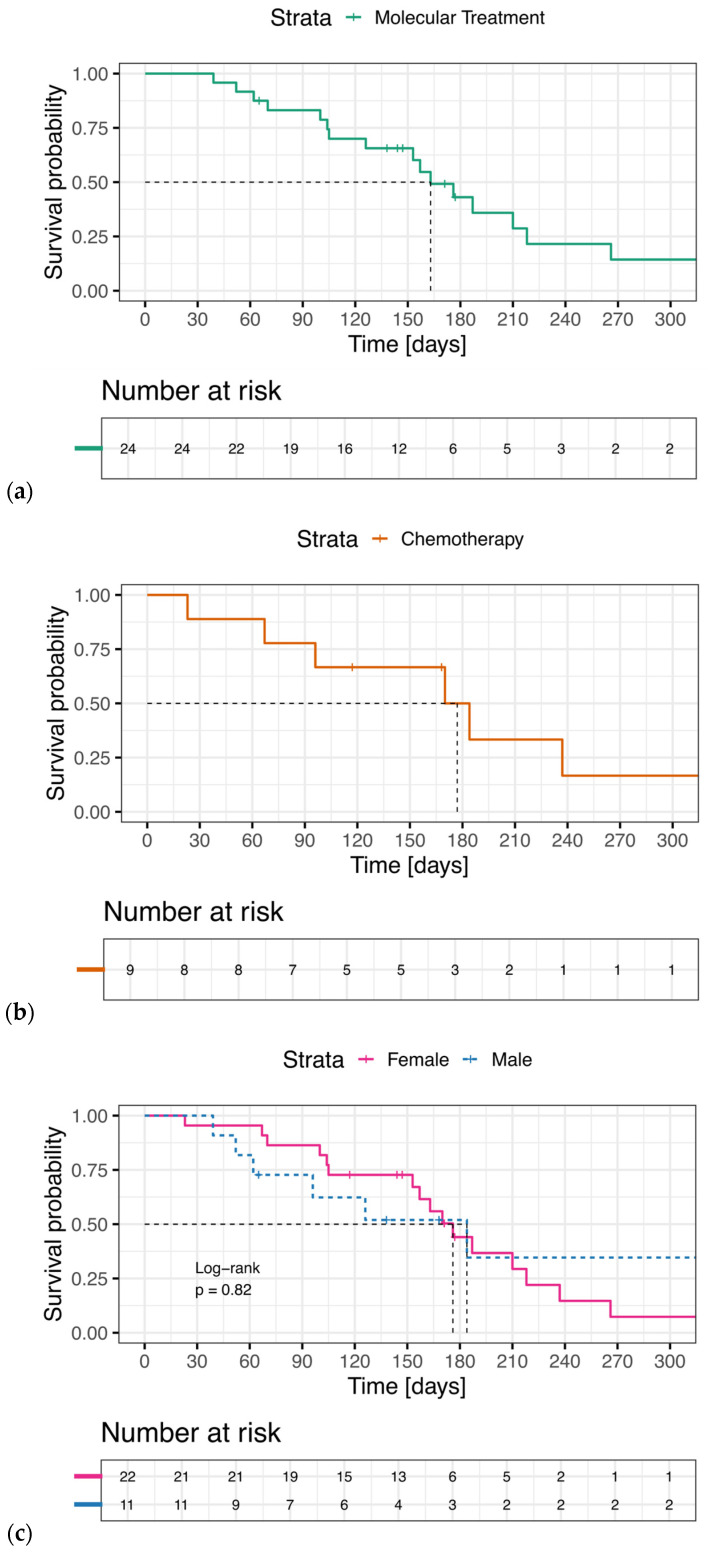
Kaplan Meier Curves of the time to imaging progression according to RECIST 1.1. (**a**) Patients receiving molecularly targeted treatment. (**b**) Patients receiving chemotherapy before starting molecularly targeted treatment. (**c**) Comparison of time to progression of patients receiving molecularly targeted treatment, stratified by gender.

**Figure 5:**
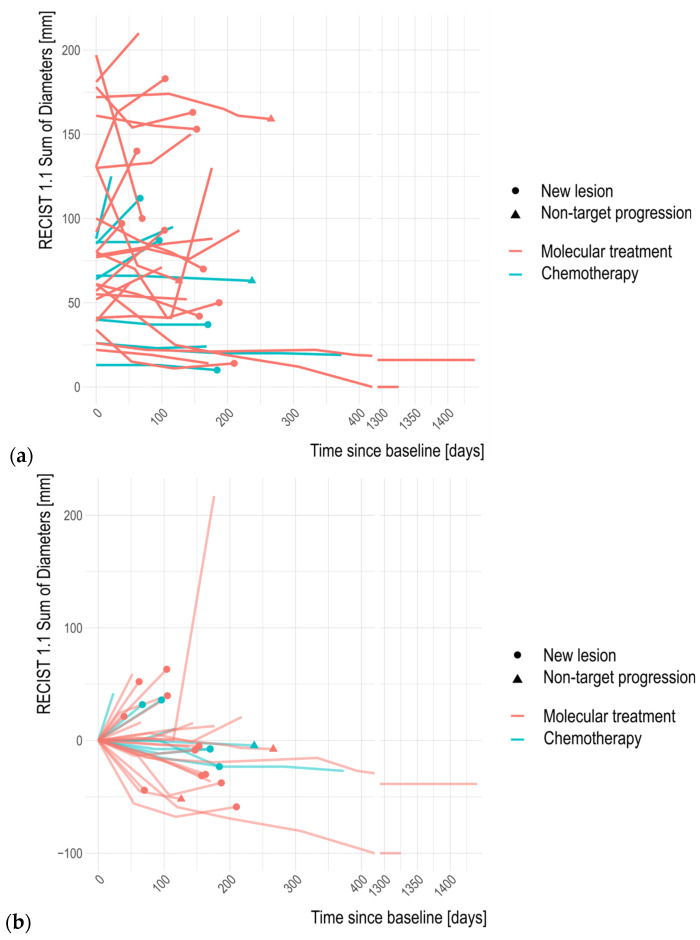
Evolution of the tumor burden of individual patients during the follow-up period. Scans marked in red denote patients receiving molecular treatment, scans in blue denote patients receiving chemotherapy. Shapes indicate the type of progression. (**a**) Percentage change in the sum of diameters and (**b**) change in the absolute sum of diameters.

In patients progressing during the follow-up period (16/24), progression occurred as target progression in 63%, nontarget progression in 13%, and appearance of new lesions in 63%. Patients could experience multiple forms of progression simultaneously (Figure 3, Figure 4 and Figure 5).

Progression according to RECIST 1.1 occurred after a median of 23 weeks. The median time to progression was comparable for male and female patients (26 and 25 weeks, respectively; *p* = 0.82; Figure 4).

## 4. Discussion

In this study, we retrospectively analyzed the metastatic patterns and implemented a standardized response assessment according to RECIST1.1 in patients with inoperable CCA referred to an MTB for personalized cancer treatment. Among 583 patients referred to the MTB, 92 were treated for CCA. Recommendations based on molecular profiles were create for 61 patients, resulting in personalized treatment options. The identification of molecular treatment targets was positively associated with the presence of liver metastasis and a higher metastatic burden in the liver and lung. Adrenal metastases were found more frequently in patients with a genetic target for PI-3 kinase inhibition. Objective responses were observed in 25% of the patients receiving personalized treatments. Additionally, personalized treatments showed potential for disease stabilization. The retrospective study emphasizes the significance of MTB for evidence-based treatment decisions and underscores the importance of whole-body imaging in monitoring personalized cancer treatment effectiveness and response assessment.

The new ESMO guidelines have firmly established the role of genotyping in patients with advanced biliary tract cancer [10]. Actionable targets, genetic alterations for which target-specific treatment options are available, are observed in nearly 40% of patients [29]. Targeted treatment for patients with *IDH1/2* mutations (10–20% of patients) [30], FGFR2 fusions and rearrangements (10–16%) [13], human epidermal growth factor receptor 2 (HER2)-amplification [31], and activating *BRAF* mutations (5%) [32] were shown to be beneficial in multiple trials. For example, Goyal et al. showed that *FGFR2*-targeted treatments benefit patients even after extensive prior treatment [14].

Zhang et al. discuss comprehensive genomic profiling (CGP) for patients with biliary tract cancer (BTC). The analysis is based on discussions at an MTB, highlighting the increasing adoption of CGP and personalized therapy in the BTC. Integrated into patient care, CGP leads to successful targeted treatments for some individuals with actionable genetic changes. The study emphasizes the need for expert guidance, e.g., through an MTB, to interpret CGP data effectively, especially given the fast-paced developments in precision oncology. For patients with advanced CCA, CGP is recommended at the initial diagnosis to offer the most suitable treatments, considering the limited standard-of-care options available [33].

Tomczak et al. have previously conducted a retrospective study of patients with CCA treated by an MTB [34]. They found actionable molecular targets in 59% of 101 patients. This is similar to our study, where treatment targets were identified in 66% of patients. In our study, 36% of those patients received a molecularly targeted treatment, similar to the investigation by Tomczak et al. (32%). Many of the treatment targets were shared between the two studies, including alterations in FGFR2, IDH, BRCA1, and PI-3 kinase. Tomczak et al. do not state which treatment response criteria were used. However, they describe observing a complete response in one patient and a partial response in five patients, resulting in an objective response rate of 30%. In this study, one patient experienced a complete response, and five patients experienced a partial response, resulting in an objective response rate of 25%. Disease stabilization was observed in 50% of the patients in our study and 30% in the study by Tomczak et al.

Typically, it takes several weeks after starting therapy to observe any noticeable changes in tumor size, which can result in patients receiving ineffective, harmful, or costly treatments before such changes become evident. The RECIST guideline simplifies image interpretation by using linear measurements as a substitute for volumetry. Modern antiangiogenic and targeted drugs prioritize stabilizing tumor growth rather than causing shrinkage, making it challenging to detect substantial volume changes [35]. Tumors like those found in the biliary tract pose challenges owing to their invasive growth pattern and irregular blood supply. To address these, an imaging biomarker that can accurately assess and monitor treatment responses is needed. PET imaging, particularly metabolic imaging with [18F] FDG, has become increasingly important in cancer management, surpassing the sensitivity of dynamic CT, MRI, and MR angiography in the preoperative staging of CCA patients [35]. Studies indicate that metabolic changes in biliary tract cancer predict progression-free survival (PFS) and overall survival, aiding treatment strategy selection [36]. A recent meta-analysis involving 2125 patients from 47 studies suggests that using [18F] FDG-PET can be valuable for staging (lymph nodes and distant metastases) and identifying recurrences in selected CCA patients [19]. Owing to the high frequency of newly appearing tumor lesions without contemporaneous progression of target lesions, the increased sensitivity of [18F] FDG-PET/CT may afford it a role in the follow-up of CCA. The uptake of [18F] FDG into primary CCA and hepatic metastases, but not lymph node metastases, predicts disease-free and overall survival [37,38]. However, data on the ability of [18F] FDG-PET/CT to predict treatment response and its incremental benefit over conventional imaging is scarce.

This study has several limitations. First, the dataset is of moderate size, and treatments were heterogeneous. MTBs receive referrals for diverse advanced tumors, and variable treatments are inherent to personalized cancer care. Second, the retrospective design of the study and its focus on two cancer centers may have introduced bias. Given that only a proportion of the patients received the recommended treatment, careful consideration and cautious interpretation of the data are necessary. Notably, all patients undergoing CGP are presented at the MTB, potentially introducing a bias towards younger and healthier individuals, which might not represent the broader CCA population and could lead to survival bias.

## 5. Conclusions

The study emphasizes the importance of incorporating MTBs into CCA treatment to provide personalized, evidence-based therapies based on genomic profiling, especially when standard treatments are ineffective. Targetable mutations, found in a significant portion of patients, were linked to specific metastatic patterns and disease progression in imaging, suggesting that genetic data should guide treatment decisions and follow-up strategies. The diverse treatment responses highlight the need for continual monitoring and adaptive treatment plans, reinforcing the value of precision medicine in improving outcomes for CCA patients.

## Figures and Tables

**Table 1:** Participant and tumor characteristics.

Characteristic		
Patients referred for CCA	92	
Age at baseline (mean ± SD)	60.3 ± 11.2 years	
Gender [F/M]	47/55	
Patients with baseline imaging	81	
Metastatic sites: Liver	0 metastases:	19 patients
	1 metastasis:	11 patients
	2–4 metastases:	17 patients
	5–9 metastases:	7 patients
	≥10 metastases:	27 patients
Lung	0 metastases:	62 patients
	1 metastasis:	0 patients
	2–4 metastases:	8 patients
	5–9 metastases:	0 patients
	≥10 metastases:	11 patients
Lymph node	0 metastases:	59 patients
	1 metastasis:	6 patients
	2–4 metastases:	6 patients
	5–9 metastases:	2 patients
	≥10 metastases:	8 patients
Peritoneum		18 patients
Pleura		4 patients
Bone		5 patients
Ovary		5 patients
Sum of diameters (mean ± SD)	81 ± 60 mm	
Molecular treatment targets		
None	27	
YAP-Inhibitor	1	
FGFR-Inhibitor	13	
TKI	9	
CDK4/6-Inhibitor	2	
PARP-Inhibitor	10	
ATR-Inhibitor	1	
VEGFR-Inhibitor	1	
Immune checkpoint Inhibitor	6	
MEK-Inhibitor	3	
IDH-Inhibitor	6	
PI3-Kinase Inhibitor	2	
MET-Inhibitor	1	
HER2-Inhibitor	2	
BRAF-Inhibitor	1	

ATR: ataxia telangiectasia and Rad3-related, BRAF: B-rapidly accelerated fibrosarcoma, CCA: cholangiocarcinoma, CDK: cyclin-dependent kinase, FGFR: fibroblast growth factor receptor, HER2: human epidermal growth factor receptor 2, IDH: isocitrate dehydrogenase, MEK: mitogen-activated protein kinase, MET: hepatocyte growth factor receptor, PARP: poly (ADP-ribose) polymerase, PI3: phosphoinositide 3, SD: standard deviation, TKI: tyrosine kinase inhibition, VEGFR: vascular endothelial growth factor receptor, YAP: yes-associated protein.

## Data Availability

The anonymized data processed for this study is made available to researchers upon reasonable request.

## Supplementary Materials

The following supporting information can be downloaded at: https://www.mdpi.com/article/10.3390/jpm14121143/s1, Table S1. Details of all patients referred to the molecular tumor board for cholangiocarcinoma. Table S2. List of patients receiving molecularly targeted treatment following a referral to the MTBs. The table details the compound recommended for personalized treatment and the molecular rationale for its choice. The best response according the RECIST 1.1 is included for every patient. Table S3. Time point treatment response data of patients undergoing molecularly targeted treatment or intermittent chemotherapy.
