# Supplementary material for: Standardized Response Assessment in Patients with Advanced Cholangiocarcinoma Treated with Personalized Therapy
Source: J Pers Med. 2024 Dec 6;14(12):1143. doi: 10.3390/jpm14121143 (PMC11679776; doi:10.3390/jpm14121143)
Supplement: Supplementary file 1 [file jpm-14-01143-s001.zip › Supplementary Table S1_R2.pdf]

Table S1. Details of all patients referred to the molecular tumor board for cholangiocarcinoma.

Pat.Nr	Sex	Diagnosis	Imaging available	Age at referral	Metastases									Molecular Targets
					Liver (n =)	Lung (n =)	Lymph nodes (n =)	Adrenal (n =)	Pleural carcinomatosis (0/1)	Peritoneal carcinomatosis (0/1)	Retroperitoneal (n =)	Soft tissue (n =)	Bone (n =)	
1.1	W	CCA	yes	73	0	0	0	0	0	1	0	0	0	no molecular target
1.2	W	CCA	yes	40	2	0	0	0	0	0	0	0	0	YAP inhibitor
1.3	W	CCA	yes	48	10+	0	10+	0	0	0	0	0	0	FGFR inhibitor
1.4	W	CCA	yes	54	0	0	5	0	0	0	0	0	0	no molecular target
1.5	W	CCA	yes	77	3		0	0	0	0	0	0	0	no molecular target
1.6	M	CCA	no											no molecular target
1.7	M	CCA	yes	66	9	3	0	0	0	0	0	0	0	Tyrosine kinase inhibitor or CDK4/6 inhibitor
1.8	M	CCA	yes	43	10+	10+	10+	0	0	0	0	0	0	FGFR inhibitor
1.9	M	CCA	yes	65	10+	0	0	0	0	0	0	0	0	PARP inhibitor
1.10	M	CCA	yes	50	0	10+	0	0	0	0	0	0	0	ATR inhibitor

Pat.Nr	Sex	Diagnosis	Imaging available	Age at referral	Metastases									Molecular Targets
					Liver (n =)	Lung (n =)	Lymph nodes (n =)	Adrenal (n =)	Pleural carcinomatosis (0/1)	Peritoneal carcinomatosis (0/1)	Retroperitoneal (n =)	Soft tissue (n =)	Bone (n =)	
1.11	W	CCA/ HCC	yes	60	10+	0	5	0	0	0	0	0	0	VEGFR inhibitor
1.12	W	extrahep CCA	yes	75	3	10+	0	0	0	0	0	0	0	no molecular target
1.13	M	CCA	yes	63	8	0	0	0	1	0	0	0	0	PARP inhibitor
1.14	M	CCA	yes	50	0	0	0	0	0	1	0	0	0	FGFR inhibitor
1.15	W	CCA	yes	73	10+	0	0	0	0	0	0	0	0	Tyrosine kinase inhibitor
1.16	M	CCA	yes	44	1	0	1	0	0	0	0	0	0	Immune checkpoint inhibitor
1.17	W	CCA	yes	51	2	0	0	0	0	0	0	0		PARP inhibitor
1.18	W	CCA	yes	67	10+	0	0	0	0	1	0	0	0	no molecular target
1.19	M	extrahep CCA	yes	70	0	0	10+	0	0	0	2	0	2	CTLA4 inhibitor or PD-1 inhibitor
1.20	M	CCA	yes	53	5	2	3	0	0	0	0	0	0	MEK inhibitor
1.21	W	CCA/ HCC	yes	54	1	0	4	0	0	1	0	0	0	Tyrosine kinase inhibitor

Pat.Nr	Sex	Diagnosis	Imaging available	Age at referral	Metastases									Molecular Targets
					Liver (n =)	Lung (n =)	Lymph nodes (n =)	Adrenal (n =)	Pleural carcinomatosis (0/1)	Peritoneal carcinomatosis (0/1)	Retroperitoneal (n =)	Soft tissue (n =)	Bone (n =)	
1.22	M	CCA	yes	65	2	10+	0	0	0	0	0	0	1	FGFR inhibitor
1.23	W	CCA	yes	59	1	0	0	0	0	1	0	1	0	no molecular target
1.24	W	CCA	yes	63	0	0	3	0	0	0	0	0	0	Anti-PD1 antibody
1.25	M	CCA	yes	78	1	0	0	2	0	0	0	0	0	Immune checkpoint inhibitor
1.26	W	CCA	yes	58	10+	2	0	0	0	0	0	0	0	FGFR inhibitor
1.27	W	CCA	yes	66	1	0	0	0	0	0	0	0	0	FGFR inhibitor
1.28	M	CCA	yes	56	4	0	0	0	0	0	0	0	0	no molecular target
1.29	W	CCA	no											IDH2 inhibitor
1.30	M	CCA/ HCC	no											no molecular target
1.31	M	CCA	yes	44	0	0	0	0	0	0	1	0	0	no molecular target
1.32	M	CCA	no											no molecular target

Pat.Nr	Sex	Diagnosis	Imaging available	Age at referral	Metastases									Molecular Targets
					Liver (n =)	Lung (n =)	Lymph nodes (n =)	Adrenal (n =)	Pleural carcinomatosis (0/1)	Peritoneal carcinomatosis (0/1)	Retroperitoneal (n =)	Soft tissue (n =)	Bone (n =)	
1.33	M	CCA	yes	70	1	0	1	0	0	0	0	0	0	no molecular target
1.34	W	CCA	yes	60	4	0	0	0	0	0	0	0	0	IDH1 inhibitor
1.35	M	CCA	yes	50	1	0	0	0	0	0	0	0	0	no molecular target
1.36	M	CCA	yes	63	1	0	0	0	0	1	0	0	0	no molecular target
1.37	W	CCA	yes	63	10+	0	0	0	0	0	0	0	0	FGFR inhibitor
1.38	M	CCA	yes	65	1	0	0	0	0	0	0	0	0	Immune checkpoint inhibitor
1.39	W	CCA	yes	57	2	0	0	0	0	1	0	0	0	PI3K inhibitor
1.40	M	CCA	yes	57	10+	0	0	0	0	10	0	0	1	CDK4/6 inhibitor
1.41	W	CCA	yes	59	10+	0	0		0	0	0	0	0	FGFR inhibitor
1.42	W	CCA	no											no molecular target
1.43	M	CCA	yes	59	10+	10+	0	0	0	0	0	0	0	FGFR inhibitor
1.44	M	CCA	yes	63	10+	2	0	0	0	0	0	0	1	PARP inhibitor

Pat.Nr	Sex	Diagnosis	Imaging available	Age at referral	Metastases									Molecular Targets
					Liver (n =)	Lung (n =)	Lymph nodes (n =)	Adrenal (n =)	Pleural carcinomatosis (0/1)	Peritoneal carcinomatosis (0/1)	Retroperitoneal (n =)	Soft tissue (n =)	Bone (n =)	
1.45	W	CCA	yes	67	10+	0	1	0	0	0	0	0	0	FGFR inhibitor
1.46	W	CCA	yes	53	10+	0	0	0	0	1	0	0	0	IDH1 inhibitor
1.47	W	CCA	no											FGFR inhibitor
1.48	M	extrahep CCA	yes	61	0	0	0	0	0	0	0	0	0	no molecular target
1.49	W	CCA	yes	58	10+	0	0	0	0	0	0	0	0	Tyrosine kinase inhibitor
1.50	M	CCA	yes	78	2	0	0	0	0	0	0	0	0	no molecular target
1.51	W	CCA	no											no molecular target
1.52	W	CCA	yes	58	10+	0	0	0	0	0	0	0	0	FGFR inhibitor
1.53	M	CCA	yes	68	1	0	0	0	1	0	0	0	0	FGFR inhibitor
1.54	W	CCA	yes	46	10+	2	5	0	0	0	0	0	0	MET inhibitor
1.55	M	CCA	yes	80	2	0	0	0	0	0	0	0	0	IDH1 inhibitor
1.56	W	CCA	yes	37	10+	10+	10+	0	1	1	0	1	0	no molecular target

Pat.Nr	Sex	Diagnosis	Imaging available	Age at referral	Metastases									Molecular Targets
					Liver (n =)	Lung (n =)	Lymph nodes (n =)	Adrenal (n =)	Pleural carcinomatosis (0/1)	Peritoneal carcinomatosis (0/1)	Retroperitoneal (n =)	Soft tissue (n =)	Bone (n =)	
1.57	M	CCA	yes	62	2	0	0	0	0	0	0	0	0	Anti-HER2 directed treatment
1.58	M	CCA	yes	80	5	0	0	0	0	0	0	0	0	Anti-HER2 directed treatment
1.59	M	CCA	no											no molecular target
1.60	W	CCA	yes	69	10+	0	0	0	0	0	0	0	0	IDH1 inhibitor
1.61	W	extrahep CCA	yes	65	0	0	2	0	0	0	0	0	0	PARP inhibitor
1.62	M	CCA	yes	69	4	0	0	0	0	0	0	0	0	PARP inhibitor
1.63	M	CCA	yes	70	0	10+	0	0	0	0	0	0	0	no molecular target
1.64	M	CCA	yes	83	0	0	0	0	0	1	0	1	0	no molecular target
1.65	W	CCA	yes	74	0	2	1	0	0	0	0	0	0	IDH1 inhibitor
1.66	W	extrahep CCA	yes	62	10+	0	0	0	0	0	0	0	0	no molecular target
1.67	M	CCA	yes	80	0	10+	0	0	0	0	0	0	0	no molecular target

Pat.Nr	Sex	Diagnosis	Imaging available	Age at referral	Metastases									Molecular Targets
					Liver (n =)	Lung (n =)	Lymph nodes (n =)	Adrenal (n =)	Pleural carcinomatosis (0/1)	Peritoneal carcinomatosis (0/1)	Retroperitoneal (n =)	Soft tissue (n =)	Bone (n =)	
1.68	W	CCA	yes	44	0	0	0	0	0	1	1	0	0	FGFR inhibitor
1.69	M	CCA	yes	48	9	0	10+	0	0	0	0	0	2	Tyrosine kinase inhibitor
1.70	W	CCA	yes	49	10+	2	10+	0	0	1	0	0	0	IDH1 inhibitor
1.71	W	CCA	yes	57	7	10+	0	0	0	1	0	0	0	PARP inhibitor
1.72	W	extrahep CCA	yes	62	0	0	0	0	0	1	0	0	0	no molecular target
1.73	M	CCA	yes	64	4	0	0	0	0	0	1	0	0	no molecular target
1.74	W	CCA	yes	41	4	0	3	0	0	0	0	0	0	PARP inhibitor
1.75	W	CCA	yes	49	1	0	1	0	0	1	0	0	0	IDH1 inhibitor
1.76	W	CCA	yes	41	0	0	2	0	0	0	0	0	0	no molecular target
1.77	W	CCA	yes	66	10+	10+	0	0	0	0	0	0	0	Tyrosine kinase inhibitor
1.78	M	CCA	yes	70	4	0	0	0	0	0	0	0	0	Tyrosine kinase inhibitor

Pat.Nr	Sex	Diagnosis	Imaging available	Age at referral	Metastases									Molecular Targets
					Liver (n =)	Lung (n =)	Lymph nodes (n =)	Adrenal (n =)	Pleural carcinomatosis (0/1)	Peritoneal carcinomatosis (0/1)	Retroperitoneal (n =)	Soft tissue (n =)	Bone (n =)	
1.79	W	CCA	yes	65	10+	3	10+	0	0	0	0	0	0	PARP inhibitor
1.80	W	CCA	yes	56	10+	0	0	0	0	0	0	0	0	BRAF inhibitor or MEK inhibitor
1.81	W	CCA	yes	58	2	0	0	0	0	0	0	0	0	no molecular target
1.82	M	extrahep CCA	no											Anti-HER2 directed treatment
1.83	M	CCA	yes	71	10+	0	0	0	0	1	0	0	0	Tyrosine kinase inhibitor
1.84	W	CCA	yes	65	0	0	0	0	0	1	0	2	0	MEK inhibitor
1.85	M	CCA	yes	35	10+	10+	10+	0	1	1	0	0	0	no molecular target
1.86	M	CCA	yes	56	10+	0	0	0	0	0	0	0	10+	no molecular target
1.87	W	CCA	no											PARP inhibitor
1.88	W	CCA	yes	60	0	0	0	0	0	0	0	0	0	no molecular target

Pat.Nr	Sex	Diagnosis	Imaging available	Age at referral	Metastases									Molecular Targets
					Liver (n =)	Lung (n =)	Lymph nodes (n =)	Adrenal (n =)	Pleural carcinomatosis (0/1)	Peritoneal carcinomatosis (0/1)	Retroperitoneal (n =)	Soft tissue (n =)	Bone (n =)	
1.89	M	CCA	yes	70	7	0	0	0	0	0	0	0	0	Tyrosine kinase inhibitor
1.90	M	CCA	yes	70	4	0	0	0	0	0	0	0	0	PARP inhibitor
1.91	M	CCA	yes	58	8	0	0	0	0	0	0	0	0	no molecular target
1.92	M	CCA	yes	36	0	0	1	1	0	0	0	1	0	PI3K inhibitor

ATR: Ataxia telangiectasia and Rad3-related, BRAF: B- Rapidly Accelerated Fibrosarcoma, CCA: Cholangiocarcinoma, CDK: Cyclin-dependent kinase, FGFR: Fibroblast growth factor receptor, HCC: Hepatocellular carcinoma, HER2: human epidermal growth factor receptor 2, IDH: MEK: Mitogen-activated protein kinase kinase, MET: hepatocyte growth factor receptor, PARP: Poly (ADP-ribose) polymerase, PI3: Phosphoinositide 3, SD: Standard deviation, TKI: tyrosine kinase inhibition, VEGFR: Vascular endothelial growth factor receptor, YAP: Yes-associated protein.
