# Supplementary material for: Standardized Response Assessment in Patients with Advanced Cholangiocarcinoma Treated with Personalized Therapy
Source: J Pers Med. 2024 Dec 6;14(12):1143. doi: 10.3390/jpm14121143 (PMC11679776; doi:10.3390/jpm14121143)
Supplement: Supplementary file 1 [file jpm-14-01143-s001.zip › Supplementary Table S2_R2.pdf]

Table S2. List of patients receiving molecularly targeted treatment following a referral to the MTBs. The table details the compound recommended for personalized treatment and the molecular rationale for its choice. The best response according the RECIST 1.1 is included for every patient.

Patient number	Molecular treatment	Rationale	Best response
1.11	FOLFOX + Bevacizumab	*TP53-Mutation. Correlation between TP53 mutations and improved response to VEGF/VEGFR inhibition.*	SD
1.15	Lenvatinib	*FGFR2* and *FGFR3* overexpression, FGFR inhibition	SD
1.19	Pembrolizumab	High mutational burden, recommendation for an immune checkpoint inhibitor.	Lost to F/U
1.20	Trametinib	Inactivation *NF1*, MEK inhibition effective in other tumor entities	PD
1.21	Lenvatinib	*FGF* amplification, FGFR inhibition	SD
1.24	Pembrolizumab	*High mutational burden and MSI-high*	PR
1.27	Pemigatinib	*FGFR2 - RBFOX2 Fusion*	SD
1.34	Ivosidenib	Missense mutation *IDH1*, CharIDHy Trial showing improved progression-free survival compared to placebo	SD
1.38	Pembrolizumab	*High mutational burden*	CR
1.43	Lenvatinib	*FGFR-TACC Fusion*	Lost to F/U
1.44	Olaparib	*BRCA2 deletion, synthetic lethality by PARP-inhibition*	PD
1.46	Ivosidenib	*IDH1* amplification, CharIDHy Trial showing improved progression-free survival compared to placebo	PD
1.49	Pemigatinib	*FGFR2-BICC1 Fusion*	PR
1.52	Cabozantinib	*FGFR2-WAC Fusion*	SD
1.54	Cabozantinib	*MET* overexpression, sensitive to Cabozantinib in case report	PD
1.60	Ivosidenib	Missense mutation *IDH1*, CharIDHy Trial showing improved progression-free survival compared to placebo	Lost to F/U
1.65	Personalized peptide vaccine	Board recommendation	PR
1.68	Pemigatinib	*FGFR2* overexpression, FGFR inhibition	Lost to F/U
1.69	Lenvatinib	Oncogenic variant of *FGFR2*, sensitive to Lenvatinib in case report.	SD
1.74	Verteporfin	*YAP1 Amplification, YAP inhibition*	PD
1.77	Lenvatinib	Oncogenic variant of *FGFR2*, sensitive to Lenvatinib in case report.	SD
1.80	Dabrafenib	*BRAF* V600E variant, BRAF inhibiton	SD

Patient number	Molecular treatment	Rationale	Best response
2.1	Pemigatin ib	*FGFR2* overexpression, FGFR inhibition	PR
2.2	Ivosidenib	*IDH1* amplification, CharIDHy Trial showing improved progression-free survival compared to placebo	SD
2.3	Pemigatinib	Overexpression of *FGFR1-4*, FGFR inhibition	SD
2.4	Ivosidenib	Missense mutation *IDH1*, CharIDHy Trial showing improved progression-free survival compared to placebo	PD
2.5	Pembrolizumab	Recommendation to start immunotherapy.	SD
2.6	Ivosidenib	*IDH1* amplification, CharIDHy Trial showing improved progression-free survival compared to placebo	PD

CR: complete response, PD: progressive disease, PR: partial response, SD: stable disease.
