# Supplementary material for: Standardized Response Assessment in Patients with Advanced Cholangiocarcinoma Treated with Personalized Therapy
Source: J Pers Med. 2024 Dec 6;14(12):1143. doi: 10.3390/jpm14121143 (PMC11679776; doi:10.3390/jpm14121143)
Supplement: Supplementary file 1 [file jpm-14-01143-s001.zip › Supplementary Table S3_R2.pdf]

Table S3. Time point treatment response data of patients undergoing molecularly targeted treatment or intermittent chemotherapy.

Patient number	Time point	Molecular therapy	Sex	Days since baseline	Modality	RECIST time point response	Sum of target diameters	%-change since baseline	%-change to nadir	New lesion	Non-target progression
CH1	0	0	m	0	CT		64	0		0	0
CH1	1	0	m	96	CT	PD	87	35.9	35.9	1	0
CH2	0	0	f	0	CT		85	0		0	0
CH2	1	0	f	67	CT	PD	112	31.8	31.8	1	0
CH3	0	0	m	0	CT		26	0		0	0
CH3	1	0	m	97	CT	SD	22	-15.4	-15.4	0	0
CH3	2	0	m	189	CT	SD	20	-23.1	-9.1	0	0
CH3	3	0	m	283	CT	SD	20	-23.1	0	0	0
CH3	4	0	m	373	CT	SD	19	-26.9	-5	0	0
CH4	0	0	f	0	CT		66	0		0	0
CH4	1	0	f	61	CT	SD	66	0	0	0	0
CH4	2	0	f	237	CT	PD	63	-4.5	-4.5	0	1
CH5	0	0	f	0	CT		88	0		0	0
CH5	1	0	f	23	CT	PD	125	42	42	0	0
CH6	0	0	f	0	CT		40	0		0	0
CH6	1	0	f	85	CT	SD	37	-7.5		0	0
CH6	2	0	f	170	CT	PD	37	-7.5	0	1	0
CH7	0	0	m	0	CT		13	0		0	0
CH7	1	0	m	93	CT	SD	13	0		0	0

Patient number	Time point	Molecular therapy	Sex	Days since baseline	Modality	RECIST time point response	Sum of target diameters	%-change since baseline	%-change to nadir	New lesion	Non-target progression
CH7	2	0	m	184	CT	PD	10	-23.1	-23.1	1	0
CH8	0	0	f	0	PET-CT		86	0		0	0
CH8	1	0	f	64	CT	SD	86	0	0	0	0
CH8	2	0	f	117	CT	SD	95	10.5	10.5	0	0
CH9	0	0	m	0	CT		26	0		0	0
CH9	1	0	m	92	CT	SD	23	-11.5	-11.5	0	0
CH9	2	0	m	168	CT	SD	24	-7.7	4.3	0	0
1.11	0	1	f	0	CT		161	0.0		0	0
1.11	1	1	f	89	CT	SD	155	-3.7	-3.7	0	0
1.11	2	1	f	153	CT	PD	153	-5.0	-1.3	1	0
1.15	0	1	f	0	PET-CT		77	0		0	0
1.15	1	1	f	78	PET-CT	SD	82	6.5	6.5	0	0
1.15	2	1	f	142	PET-CT	SD	76	-1.3	-1.3	0	0
1.15	3	1	f	218	PET-CT	PD	93	20.8	22.4	0	0
1.2	0	1	m	0	CT		80	0		0	0
1.2	1	1	m	39	CT	PD	97	21.3	21.3	1	0
1.21	0	1	f	0	CT		172	0		0	0
1.21	1	1	f	111	CT	SD	174	1.2	1.2	0	0
1.21	2	1	f	194	CT	SD	165	-4.1	-4.1	0	0
1.21	3	1	f	216	CT	SD	161	-6.4	-2.4	0	0

Patient number	Time point	Molecular therapy	Sex	Days since baseline	Modality	RECIST time point response	Sum of target diameters	%-change since baseline	%-change to nadir	New lesion	Non-target progression
1.21	4	1	f	266	CT	PD	159	-7.6	-1.2	0	1
1.24	0	1	f	0	PET-CT		26	0.0		0	0
1.24	1	1	f	76	PET-CT	SD	22	-15.4	-15.4	0	0
1.24	2	1	f	175	PET-CT	SD	21	-19.2	-4.5	0	0
1.24	3	1	f	333	PET-CT	SD	22	-15.4	4.8	0	0
1.24	4	1	f	394	CT	SD	19	-26.9	-9.5	0	0
1.24	5	1	f	493	CT	PR	17	-34.6	-10.5	0	0
1.24	6	1	f	590	CT	PR	17	-34.6	0	0	0
1.24	6	1	f	687	CT	PR	20	-23.1	17.6	0	0
1.24	6	1	f	791	CT	PR	18	-30.8	5.9	0	0
1.24	6	1	f	842	PET-CT	PR	16	-38.5	-5.9	0	0
1.24	6	1	f	974	PET-CT	PR	14	-46.2	-12.5	0	0
1.24	6	1	f	1066	CT	PR	16	-38.5	14.3	0	0
1.24	6	1	f	1254	CT	PR	16	-38.5	14.3	0	0
1.24	7	1	f	1441	CT	PR	16	-38.5	14.3	0	0
1.27	0	1	f	0	CT		61	0.0		0	0
1.27	1	1	f	63	CT	SD	55	-9.8	-9.8	0	0
1.27	2	1	f	157	CT	PD	42	-31.1	-23.6	1	0
1.34	0	1	f	0	PET-CT		41	0		0	0
1.34	1	1	f	56	PET-CT	SD	42	2.4	2.4	0	0

Patient number	Time point	Molecular therapy	Sex	Days since baseline	Modality	RECIST time point response	Sum of target diameters	%-change since baseline	%-change to nadir	New lesion	Non-target progression
1.34	2	1	f	114	PET-CT	SD	41	0	0	0	0
1.34	3	1	f	176	PET-CT	PD	130	217.1	217.1	0	0
1.38	0	1	m	0	CT/MRT		61	0.0		0	0
1.38	1	1	m	120	CT/MRT	PR	25	-59.0	-59	0	0
1.38	2	1	m	197	CT/MRT	PR	19	-68.9	-24	0	0
1.38	3	1	m	308	CT	PR	12	-80.3	-36.8	0	0
1.38	4	1	m	420	CT	CR	0	-100.0	-100	0	0
1.38	5	1	m	542	CT	CR	0	-100.0	0	0	0
1.38	6	1	m	634	CT	CR	0	-100.0	0	0	0
1.38	7	1	m	744	CT	CR	0	-100.0	0	0	0
1.38	8	1	m	844	CT	CR	0	-100.0	0	0	0
1.38	9	1	m	939	CT	CR	0	-100.0	0	0	0
1.38	10	1	m	1037	CT	CR	0	-100.0	0	0	0
1.38	11	1	m	1134	CT	CR	0	-100.0	0	0	0
1.38	12	1	m	1319	CT	CR	0	-100.0	0	0	0
1.38	14	1	m	1325	CT	CR	0	-100.0	0	0	0
1.44	0	1	m	0	PET-CT		92	0		0	0
1.44	1	1	m	62	PET-CT	PD	140	52.2	52.2	1	0
1.46	0	1	f	0	CT		131	0		0	0
1.46	1	1	f	32	CT	PD	163	24.4	24.4	0	0

Patient number	Time point	Molecular therapy	Sex	Days since baseline	Modality	RECIST time point response	Sum of target diameters	%-change since baseline	%-change to nadir	New lesion	Non-target progression
1.49	0	1	f	0	PET-CT		34	0.0		0	0
1.49	1	1	f	54	PET-CT	PR	15	-55.9	-55.9	0	0
1.49	2	1	f	118	PET-CT	PR	11	-67.6	-26.7	0	0
1.49	3	1	f	210	CT	PD	14	-58.8	27.3	1	0
1.52	0	1	f	0	CT		80	0.0		0	0
1.52	1	1	f	59	CT	SD	70	-12.5	-12.5	0	0
1.52	2	1	f	108	CT	PR	41	-48.8	-41.4	0	0
1.52	3	1	f	187	CT	PD	50	-37.5	22	1	0
1.54	0	1	f	0	PET-CT		197	0		0	0
1.54	1	1	f	70	PET-CT	PD	100	-44.1	-49.2	1	0
1.65	0	1	f	0	CT		22	0		0	0
1.65	1	1	f	84	CT	SD	19	-13.6	-13.6	0	0
1.65	2	1	f	171	PET-CT	PR	14	-36.4	-26.3	0	0
1.69	0	1	m	0	PET-CT		181	0		0	0
1.69	1	1	m	65	CT	SD	210	16	16	0	0
1.74	0	1	f	0	CT/MRT		52	0.0		0	0
1.74	1	1	f	100	CT/MRT	PD	71	36.5	36.5	0	0
1.77	0	1	f	0	PET-CT		178	0		0	0
1.77	1	1	f	55	PET-CT	SD	154	-13.5	-13.5	0	0
1.77	2	1	f	147	CT	SD	163	-8.4	5.8	1	0

Patient number	Time point	Molecular therapy	Sex	Days since baseline	Modality	RECIST time point response	Sum of target diameters	%-change since baseline	%-change to nadir	New lesion	Non-target progression
1.8	0	1	f	0	CT		100	0		0	0
1.8	1	1	f	70	CT	SD	86	-14	-14	0	0
1.8	2	1	f	114	CT	SD	80	-20	-7	0	0
1.8	3	1	f	163	CT	PD	70	-30	-12.5	1	0
2.1	0	1	m	0	CT		131	0		0	0
2.1	1	1	m	63	CT	PR	72	-45	-45	0	0
2.1	2	1	m	126	CT	PD	63	-51.9	-12.5	0	1
2.2	0	1	f	0	CT/MRT		78	0		0	0
2.2	1	1	f	105	CT/MRT	SD	85	9	9	0	0
2.2	2	1	f	177	CT/MRT	SD	88	12.8	12.8	0	0
2.3	0	1	f	0	CT		130	0		0	0
2.3	1	1	f	84	CT	SD	133	2.3	2.3	0	0
2.3	2	1	f	144	CT	SD	150	15.4	15.4	0	0
2.4	0	1	f	0	CT		57	0		0	0
2.4	1	1	f	104	CT	PD	93	63.2	63.2	1	0
2.5	0	1	m	0	CT		55	0		0	0
2.5	1	1	m	138	CT	SD	52	-5.5	-5.5	0	0
2.6	0	1	m	0	CT		39	0		0	0
2.6	1	1	m	52	CT	PD	62	59	59	0	0

Patients receiving chemotherapy are marked with “CH”. CR: complete response, PD: progressive disease, PR: partial response, SD: stable disease.
